# Diversity and Host Specificity of Avian Haemosporidians in an Afrotropical Conservation Region

**DOI:** 10.3390/ani14192906

**Published:** 2024-10-09

**Authors:** Mduduzi Ndlovu, Maliki B. Wardjomto, Tinotendashe Pori, Tshifhiwa C. Nangammbi

**Affiliations:** 1 School of Biology and Environmental Sciences, University of Mpumalanga, Mbombela 1201, South Africa; 2 School of Life Sciences, University of Warwick, Coventry CV4 7AL, UK; 3 Department of Nature Conservation, Tshwane University of Technology, Pretoria 0001, South Africa

**Keywords:** avian malaria, diseases, diversity, parasites, prevalence

## Abstract

**Simple Summary:**

African tropical regions have a remarkably high bird diversity, yet few studies have tried to unravel the presence of blood parasites in birds found in conservation areas. Knowing which blood parasites are present will help us to prepare for potential disease outbreaks. We test the hypothesis that conservation regions have a high diversity of parasites. Molecular methods were used to screen 1035 blood samples from 55 bird species for blood infections on sites inside and adjacent to the Kruger National Park in South Africa. Overall, 28.41% of birds were found infected with at least one type of blood parasites. Bird malaria of the type *Haemoproteus* and *Plasmodium* was found in 17.39% and 4.64% of the birds respectively. *Leucocytozoon* blood parasite was found in 9.24% of birds. One hundred distinct blood parasite types were detected, of which 56 were new types. Similar bird malaria (*Haemoproteus* and *Plasmodium*) infections were found in closely related birds, while *Leucocytozoon* was found in almost every bird type. Sites with a high bird diversity also had a high parasite diversity. These findings provide insight of how birds can gradually survive their blood parasite infections.

**Abstract:**

Afrotropical regions have high bird diversity, yet few studies have attempted to unravel the prevalence of avian haemosporidia in conservation areas. The diversity and host specificity of parasites in biodiversity hotspots is crucial to understanding parasite distribution and potential disease emergence. We test the hypothesis that biodiverse regions are associated with highly diverse parasites. By targeting the cytochrome b (*Cytb*) gene, we molecularly screened 1035 blood samples from 55 bird species for avian haemosporidia infections to determine its prevalence and diversity on sites inside and adjacent to the Kruger National Park. Overall infection prevalence was 28.41%. *Haemoproteus*, *Leucocytozoon,* and *Plasmodium* presented prevalences of 17.39%, 9.24%, and 4.64%, respectively. One hundred distinct parasite lineages were detected, of which 56 were new lineages. *Haemoproteus* also presented the highest diversity compared to *Leucocytozoon* and *Plasmodium* with varying levels of specificity. *Haemoproteus* lineages were found to be specialists while *Plasmodium* and *Leucocytozoon* lineages were generalists. We also found a positive relationship between avian host diversity and parasite diversity, supporting an amplification effect. These findings provide insight data for host–parasite and co-evolutionary relationship models.

## 1. Introduction

The diversity of parasites is an important selective force shaping communities and ecosystems. Parasites generally have a higher mutation rate than their hosts, in order to evade the host immune system and successfully proliferate in an ecosystem [1]. The parasites’ ability to diversify and mutate can lead to niche expansions and pathogen host shifts allowing the infection of numerous hosts [2]. On the other hand, parasite diversity may be driven by the diversity and evolutionary life-history traits of the available hosts [3] as well as the prevailing environmental factors [4]. Changes in the environment affect hosts as well as the host–parasite relationship [5]. Host specificity is therefore a strong determinant of both parasite diversity and prevalence, which provides an opportunity to understand the mechanisms driving parasite spillovers and factors linked with emerging infectious diseases [6].

The survival and proliferation of parasites in an ecosystem is dependent on several evolutionary strategies and traits developed in relation to their host (The Red Queen hypothesis) and the habitat type where they occur. One such trait is host specificity, which represents the number and/or diversity of the host species that a parasite can infect [7]. Such a strategy can be explained by the “Trade-off” hypothesis and the “Niche-breadth” hypothesis [8]. The parasite could be a generalist, i.e., it can infect multiple host species achieving a high or low prevalence, as opposed to a specialist that will be found in only one or a few closely related species, achieving a higher prevalence than a generalist by predominantly infecting more closely related host species [9,10]. The ability of a parasite to infect a host is also dependent on the presence of susceptible hosts, competent vectors, and a permissive environment [11]. As such, a parasite that is a specialist in one ecosystem may appear to be a generalist in another and vice versa [10], a pattern that was observed with vector species as well [12]. Host switching strategies may also occur where parasites infect many host species to avoid the host’s defences [13]. In birds, a host species’ anti-parasite behaviours (i.e., body maintenance, nest maintenance, avoidance of parasitized prey using cues to their presence in conspecifics and intermediate hosts, migration, and tolerance) in combination with immune system defences may reduce a parasite load thus driving host switching by the parasite [14]. Although poorly understood, the interaction of behaviours such as preening, scratching, dusting, nest site avoidance, nest sanitation, migration, and other behavioural defences may drive a parasite to switch from one host species to another.

Specialist parasites with life cycles interdependent with that of hosts (e.g., in lice where the host represents the parasite’s only environment), usually develop a narrow level of host specificity and only infect members of a single species which in turn determines their population structure, abundance, and prevalence [15]. In such cases, a change in host ecology can either cause a proliferation or extinction of the parasite lineage. However, Medeiros et al. [16] observed that specialists can compensate for the reduced host breadth by achieving a higher prevalence in a single host species. Generalist parasites, whose life history is not exclusively limited to one specific host, benefit from a high host diversity because they can infect multiple host species, which enables them to persist in ecosystems and potentially spread to immunologically naïve hosts, i.e., hosts that have not evolved with the parasite [13]. Host specificity is an important aspect of parasite communities and should be a key component of all wildlife disease studies because it can determine the chance of survival of a parasite in the case of a host species’ extinction, the invasion potential of a parasite in new habitats such as islands, or the establishment and spread of a parasite following its introduction to a new geographical area [7].

Avian haemosporidian parasites of the genera *Plasmodium*, *Haemoproteus*, and *Leucocytozoon* are widespread vector-transmitted blood parasites that exhibit varying levels of prevalence, diversity, and host specificity at various spatiotemporal scales and across ecoregions [17]. This spatiotemporal variation could be the result of host- or parasite-mediated adaptions to the environment. Previous studies of avian haemosporidian parasites in the southern African region found varying prevalence levels, which were generally affected by the sampling effort (a low prevalence with a small sampling size and small sampling areas), while parasite lineage diversity was high as predicted [18,19,20]. Avian haemosporidia also exhibit varying levels of host preferences that may be driven by the host’s ecological traits [19,21]. While *Haemoproteus* spp. tend to be more host specific [22], *Plasmodium* spp. have generally been found to be generalists [22,23] and *Leucocytozoon* parasites exhibit varying degrees of host specificity depending on the species and the ecological context [24]. Exceptions have also been observed where certain lineages may be restricted to a specific group of birds for all three avian haemosporidian parasite genera [25].

Despite a growing number of avian haemosporidian parasites studies, certain ecoregions and bird species remain under-sampled [26]. Few large-scale studies have been conducted on avian haemosporidia in the Afrotropical regions. In the face of global environmental change and large-scale disease emergence and spread, a substantial number of studies, especially in biodiversity hotspots, are necessary to improve our understanding of disease prevalence, diversity, and disease risk mapping. This large-scale study unravels the avian haemosporidian parasite community and host specificity at an Afrotropical biodiversity conservation region. Notwithstanding the significant role of the environment, we test the hypothesis that biodiverse regions are associated with highly diverse parasite communities. In other words, a conservation region with a high variety of potential hosts will present opportunities for a diverse parasite community. We also examined the relationship between host specificity and parasite prevalence, and test the hypothesis that host specificity, parasite prevalence, and diversity are correlated.

## 2. Materials and Methods

### 2.1. Study Area

Birds were sampled from nine sites in an Afrotropical lowveld conservation area within the greater Kruger region in South Africa. The sampling was conducted in Kruger National Park (Skukuza, Satara, Phalaborwa, Shangoni, and Shingwedzi) and the surrounding settlement areas (Acornhoek, Hazyview, Mkhuhlu, and Malelane). Sampling sizes and sampling events differed between sites and seasons where certain sites were sampled only once whilst other sites were sampled repeatedly due to ease of access and availability. More than 500 bird (both resident and migrant) species were recorded in this region [27]. This biodiversity rich region is approximately 19,600 km^2^ in size; consists of three overlapping ecoregions of the world (Drakensberg montane grasslands, woodlands and forests, Southern Africa bushveld, and Zambezian and Mopane woodlands [28]; and experiences a subtropical climate (Köppen climate classification: BSh (Climate: arid; Precipitation: steppe; Temperature: hot arid)) characterised by hot wet summers (average temperature of 26.4 °C) and mild frost free dry winters (average temperature 17.8 °C). The rainy season is from September to May, with a rainfall gradient which decreases from the southern (750 mm per annum) to the northern parts of Kruger National Park (350 mm per annum).

### 2.2. Sampling Design and Protocol

**Figure 1:**
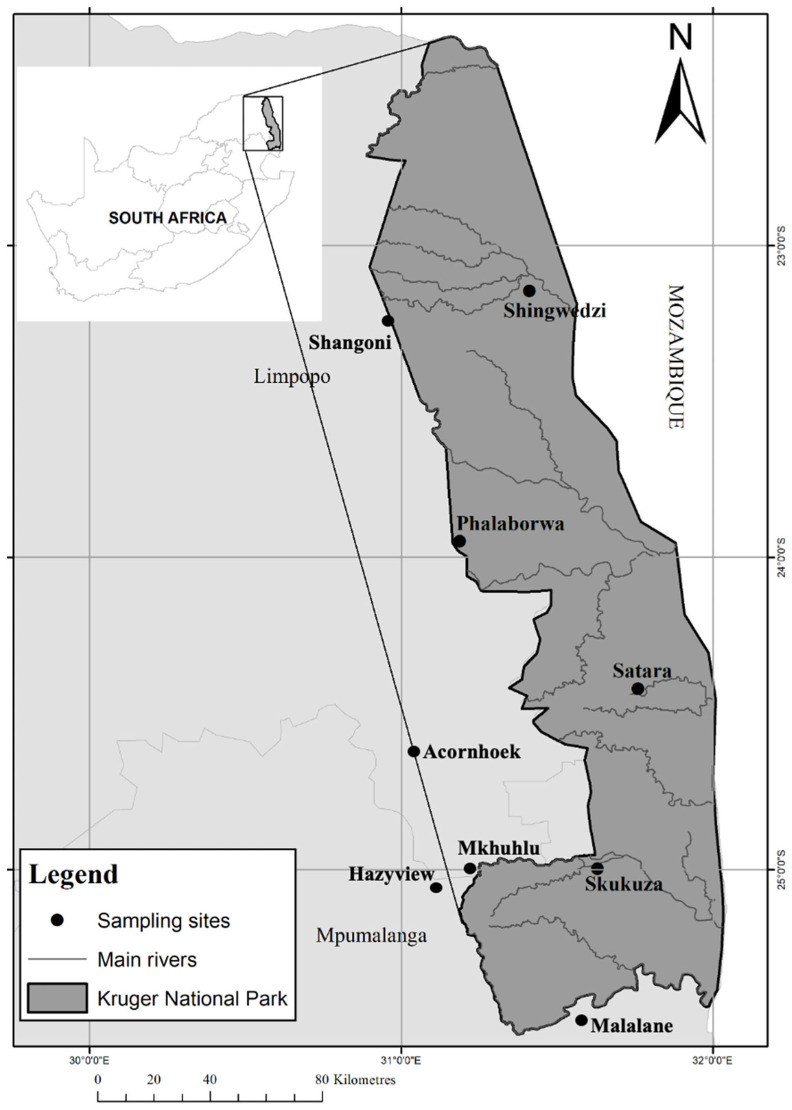
Geographic location of sampling sites inside and outside Kruger National Park.

Fieldwork was performed during the dry and wet seasons from April 2015 to November 2017. Five sampling sites were located within Kruger National Park and another four sampling sites outside the park were selected (Figure 1). Because of the nature of the park (a Big five area with security and poaching problems) and the frequent civil unrest among communities outside the park, birds were sampled opportunistically or as the sampling permits allowed and when the opportunity to visit the sites was presented. As such, certain sites were sampled only once during the three-year sampling period (Acornhoek and Malelane—2017 wet and dry) and for one single season (Hazyview—2016 wet season; Shangoni—2015 dry season; Phalaborwa—2016 dry season and 2017 wet season) whilst others were sampled for more than one year and multiple seasons were covered. Skukuza was sampled throughout the three years and during both wet and dry seasons. Satara and Shingwedzi were sampled during the wet and dry seasons of 2016 and 2017.

Live birds were sampled using birdcall lure baited mist-nets [29]. Standard morphometric measurements were taken from all captured birds (tarsus; head and culmen lengths; body mass; state of moult). Blood samples were obtained by venepuncture of the brachial vein on the right wing using a sterile 25 G needle, with blood drawn into a 75 µL micro-haematocrit capillary tube. A drop of blood was added in a vial with lysis buffer for DNA extractions and molecular detection screening. Sampled birds were released at the capture site immediately after processing.

### 2.3. Parasite Screening

To determine whether the birds harboured any avian haemosporidian parasites (genera: *Haemoproteus*, *Plasmodium*, and *Leucocytozoon*), genomic DNA was extracted from blood samples using the commercial DNeasy Blood and Tissue extraction kit (Qiagen, Valencia, CA, USA) and Invisorb Spin Blood mini kit (Stratec molecular, Berlin, Germany). Extracted DNA was quantified using the NanoDrop 2000 Spectrophotometer (Thermo Fisher Scientific, Wilmington, DE 19810 USA running the NanoDrop 2000 operating software) and then diluted to a working concentration of approximately 25 ng/µL using a TE buffer. Samples with lower DNA concentrations were not diluted. Thereafter, DNA samples were screened for haemosporidian parasites using the nested PCR protocol described by Hellgren et al. [30]. A 479 bp fragment of the parasite’s cytochrome b gene was amplified. Primer set HaemNFI and HaemNR3 were used in the first PCR to amplify the DNA of all three genera: *Plasmodium*, *Haemoproteus*, and *Leucocytozoon*. In the second PCR, we used the product of the first PCR with primer set HaemF and HaemR2 to amplify *Plasmodium* and *Haemoproteus*, and primer set HaemFL and HaemR2L for *Leucocytozoon*. A positive control (DNA template from the bird infected with *Haemoproteus*/*Plasmodium* and *Leucocytozoon*) and a negative control (distilled water) were included for every 24 samples in a 96-well plate. PCR products (1.5 µL) were checked in a 2% agarose gel stained with GelRed (Biotium, Fremont, CA, USA) using the Mupid-One electrophoresis system (Mupid Co., Ltd., Tokyo, Japan) at 50 V for 45 min and observed under UV light for bands, looking for bands of the appropriate size (479 bp). The PCR run was validated if all positive controls showed positive amplification, and the negative controls showed no amplification within the 96-well plate. Samples were run twice to confirm the results and exclude instances of false positivity or negativity. All positive PCR products were sent to Macrogen (Macrogen Inc., Amsterdam, The Netherlands) for purification and forward sequencing. Suspected new lineages were subjected to a second nested PCR protocol and sent to Macrogen for reverse sequencing to obtain the full length of the sequence.

### 2.4. Parasite Prevalence and Diversity

All statistical analyses were conducted using R version 4.0.1 [31] on its integrated development environment R Studio version 1.3.959 [32]. Infection prevalence was calculated as the proportion of infected individuals of each host species, determined per site as well as per parasite genus. Host species diversity (*H_H_*) and parasite lineage diversity (*H_P_*) of the parasite lineages infecting each host species were both calculated using Shannon’s diversity index [33] as implemented in the R package vegan version 2.6 [34]. Data were tested for normality and a Pearson correlation coefficient was used to measure the linear association between the host (bird) and parasite diversity indices. A rarefaction analysis was also carried out to evaluate the completeness of our sample diversity.

### 2.5. Phylogenetic Analyses

Sequences obtained from Macrogen were individually checked, edited, and aligned manually using BioEdit version 7.0.5.2 [35]. The resulting sequences were individually entered in the GenBank [36] and MalAvi (accessed on 26 February 2020 [37]) databases for search and identification purposes using BLAST (Basic Local Alignment Search Tool version 2.7.1 [38]). Newly identified lineages were assigned new lineage names following the MalAvi standardized nomenclature [37] whilst sequences that were a 100% match to a lineage in the databases were assigned the corresponding MalAvi lineage name. All unique parasite lineages were subjected to phylogenetic analyses. Newly recovered parasite lineages were deposited in the GenBank database (accession numbers MW546939–94) and submitted to the MalAvi database.

A phylogenetic analysis was carried out to determine the evolutionary relationships between all the unique parasite lineages detected in this study using the Maximum Likelihood method [39]. A full phylogenetic tree was also drawn. The General Time Reversible with 5 gamma distributions (GTR + G) model was determined as the best substitution model suitable for phylogenetic reconstruction by the model function in MEGA X [40], with the lowest Bayesian Information Criteria (BIC) score. The analysis made use of 1000 bootstrap replications to generate bootstrap values.

### 2.6. Host Specificity

The host specificity index $\;(S_{TD^{*}})$ described by [7] was used to determine the host specificity of the haemosporidian lineages identified in this study. The program TaxoBiodiv2 (http://www.otago.ac.nz/parasitegroup/downloads.html, accessed on 20 April 2023) was used. The index measured the average taxonomic distinctness of all host species infected by a parasite species, weighted by the prevalence of the parasite in these different hosts, and is calculated as follows:

$$S_{TD^{*}}=\frac{\sum \sum_{i<j}\omega_{ij}\left(p_{i}p_{j}\right)}{\sum \sum_{i<j}\left(p_{i}p_{j}\right)}$$

where the summations are above the set (*i* = 1, …, *s*; *j* = 1, …, *s*, such that *i* < *j* and *s* is the number of host species used by the parasite), $\omega_{ij}$ is the taxonomic distinctness between the host species *i* and *j*, and $p_{i}$ and $p_{j}$ are the prevalence of the parasite in the host species *i* and *j*, respectively [7]. In this study, the lineages that infected only one bird species were excluded from the analysis and were assigned an S_TD__*_ value of 1 as suggested by [7]. The lower values indicate parasite lineages that infect closely related hosts, while the higher values indicate parasite lineages that infect a wide range of host species.

For the host specificity index, only the lineages that infected two or more bird species were used; those detected only once were excluded since they do not provide information on the range of hosts. The construction of lineage networks for each parasite genus was performed using the medium joining network method, to test if a group of lineages or cluster of lineages were specific to certain avian families.

## 3. Results

### 3.1. Host and Parasite Diversity

**Figure 2:**
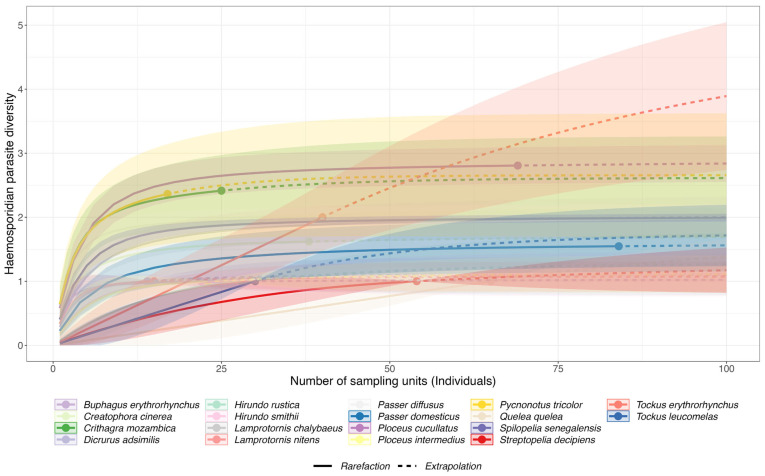
Rarefaction and extrapolation curves of the sampled birds against the haemosporidian parasite diversity.

A total of 1035 birds belonging to 55 species, 46 genera, 33 families, and 12 orders were sampled, of which 294 individuals (28.41%) were infected with at least one parasite genus including coinfections (Supplementary Table S1). The observed rarefaction curve indicated that our sampled bird numbers and diversity (except for Wire-tailed Swallow, *Hirundo smithii*) were indeed sufficient for the haemosporadian parasites detected (Figure 2).

The prevalence of *Haemoproteus* was 17.39% (*n* = 180 birds), *Leucocytozoon* was 9.28% (*n* = 96 birds), and *Plasmodium* infection was 4.64% (*n* = 48 birds, Table 1). Of the 294 infected birds, 180 were infected with *Haemoproteus* (61.22%), 48 with *Plasmodium* (16.32%), and 96 with *Leucocytozoon* (32.65%). Considering the bird species with sample sizes ≥ 30 birds, the highest infection prevalence for any parasite was in the Southern grey-headed Sparrows (*Passer diffusus*) and the Village Weaver (*Ploceus cucullatus*) at 65% and 55%, respectively. Whereas the lowest infection prevalence for any parasite was in the Red-billed Quelea (*Quelea quelea*) and Southern yellow-billed Hornbill (*Tockus leucomelas*) at 2% and 3%, respectively. Fifteen out of fifty-five bird species did not present any form of avian haemosporidian infection (Supplementary Table S1).

One hundred distinct avian haemosporadian parasite lineages were detected for which 56 were new while 44 were already in the MalAvi database (Table 1). The most prevalent lineage was RS4 (*Leucocytozoon* sp.) and it infected the highest number of birds (*n* = 28 individuals, from six different bird species).

Coinfection (defined as infections with two or more different parasites, [37]), was recorded in 28 individual birds from 15 species (12 families, namely: Columbidae, *n* = 7; Passeridae, *n* = 4; Pycnonotidae, *n* = 4; Fringillidae, *n* = 3; Ploceidae, *n* = 3; Buphagidae, *n* = 1; Lybiidae, *n* = 1; Monarchidae, *n* = 1; Muscicapidae, *n* = 1; Paridae, *n* = 1; Phasianidae, *n* = 1; and Sturnidae, *n* = 1). They comprised 18 *Haemoproteus* + *Leucocytozoon* and four *Plasmodium* + *Leucocytozoon* infection combinations. The infections by *Haemoproteus* and *Plasmodium* could not be resolved. Six cases of infections by two different lineages of *Leucocytozoon* were also recorded. Another case of multiple infections by three parasite lineages was also observed in a Laughing Dove (*Spilopelia senegalensis*) which comprised one *Haemoproteus* lineage and two *Leucytozoon* lineages.

**Figure 3:**
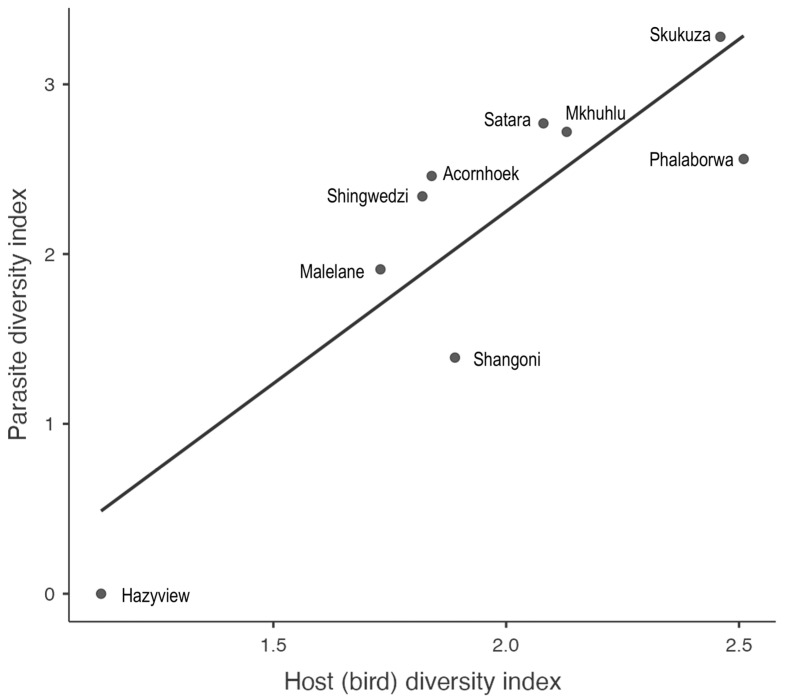
Relationship between bird diversity and parasite lineage diversity indices across the nine sampling sites inside and adjacent to Kruger National Park. Fitted linear regression: *y* = 2.028*x* – 1.805; *r* = 0.866; *F* = 21.059; *p* = 0.0025.

The calculated bird species diversity index among sites ranged from 1.13 to 2.51. Whereas the bird species richness varied between four and thirty-six (Table 2). In comparison, parasite lineage diversity indices per site ranged between 0 and 3.28. While parasite lineage richness was found to be between one and forty-six (Table 2). Overall, bird species diversity was positively correlated with parasite lineage diversity (*y* = 2.028*x* − 1.805, *r* = 0.866, *F* = 21.059, *p* = 0.0025, Figure 3).

Four *Haemoproteus* lineages (AFR041, AFR076, CRECRI01 and SPISEN02) infected the most individuals whilst three *Leucocytozoon* lineages (REB7, RS4, and SPISEN06) and one *Plasmodium* lineage (TOCERY01) were also common (Table S1). The birds infected with the highest number of parasite lineages were the Village Weavers (19 lineages), Greater blue-eared Starlings (*Lamprotornis chalybaeus*; 12 lineages), and Southern grey-headed Sparrows (12 lineages). All three are resident species.

### 3.2. Phylogenetic Relationships

**Figure 4:**
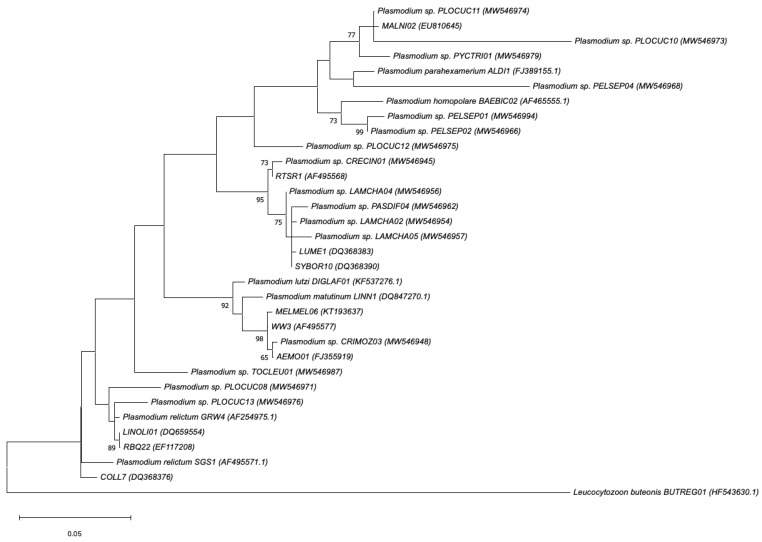
Phylogenetic relationship between *Plasmodium* parasite lineages (479 bp mitochondrial cytochrome b) detected inside and adjacent to Kruger National Park, as determined by the Maximum Likelihood (ML) method. ML bootstrap values >50% are indicated next to the branch nodes. The closest described species match from the NCBI BLAST search, for each cluster, is included in the tree. GenBank accession numbers are included in brackets.

**Figure 5:**
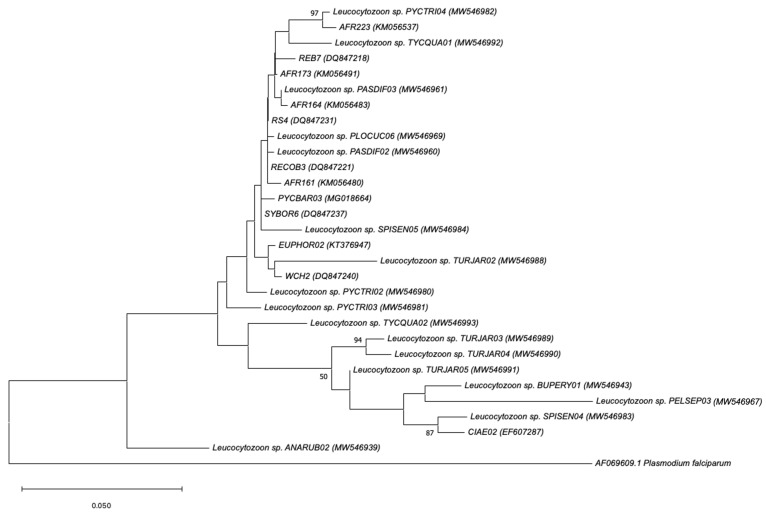
Phylogenetic relationship of *Leucocytozoon* parasite lineages (479 bp mitochondrial cytochrome b lineages) detected inside and adjacent to Kruger National Park, as determined by Maximum Likelihood (ML) method. ML bootstrap values >50% are indicated next to the branch nodes. GenBank accession numbers are included in brackets.

**Figure 6:**
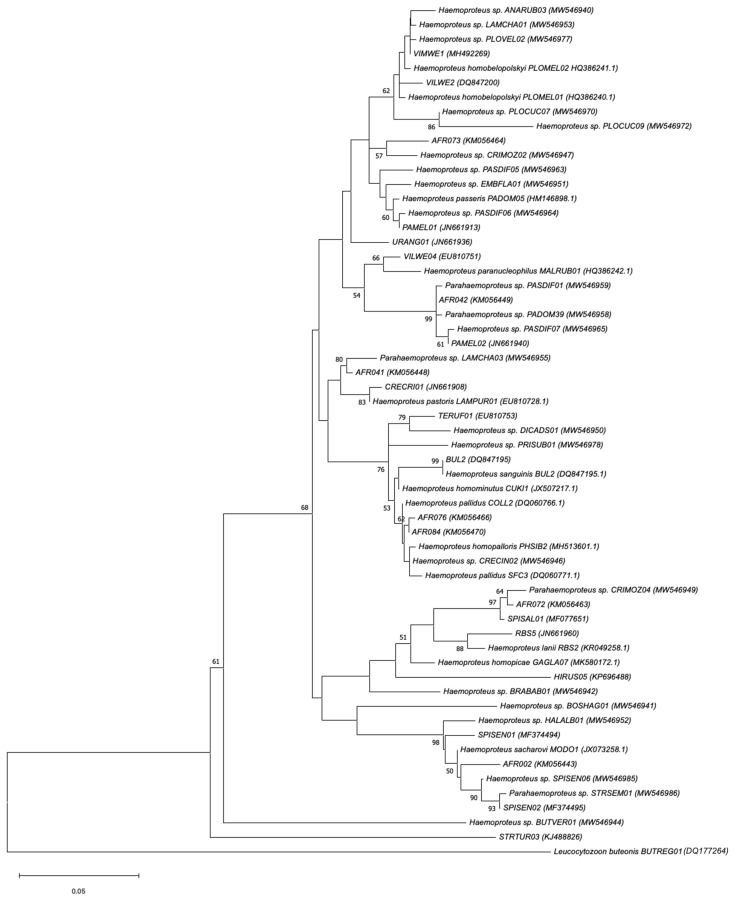
Phylogenetic relationship of *Haemoproteus* parasite lineages (479 bp mitochondrial cytochrome b lineages) detected inside and adjacent to Kruger National Park, as determined by the Maximum Likelihood (ML) method. ML bootstrap values (>50%) from 1000 replications are indicated next to the branch nodes. The closest described species match from the NCBI BLAST search for each cluster is shown. GenBank accession numbers are included in brackets.

The individual parasite genus phylogenetic trees are provided separately (in sub-trees) for better visibility of the relationship between the lineages (Figure 4, Figure 5 and Figure 6). The *Plasmodium* phylogenetic tree formed five clusters, with the largest cluster comprising eight lineages (Figure 4). The smallest cluster contained only two parasite lineages. The lineage PELSEP04, a novel parasite lineage detected from a Crested Francolin (*Peliperdix sephaena*), was unique and differed by 24 bp from its most closely related lineage in the MalAvi and Genbank databases, with a 95% similarity to the lineage TURPEL05 (GenBank accession number: MG018674.1).

The *Leucocytozoon* tree identified three clusters, including the largest cluster (with 22 parasite lineages) and two small clusters with two and three lineages (Figure 5). All 22 lineages in the largest cluster showed between 96 and 99% similarity to *Leucocytozoon gentili*. Similar to lineage PELSEP04 (*Plasmodium* sp.) described above, lineage PELSEP03 (*Leucocytozoon* sp.) was unique, and it was also found in the same individual. PELSEP03 was 95% similar to *Leucocytozoon schoutedeni*, which was previously recovered and described in chickens (lineages GALLUS06 and GALLUS07; accession numbers DQ676823 and DQ676824, respectively). Both lineages PELSEP03 and PELSEP04 were newly identified lineages in this study and were a coinfection case in the same bird and the first record in this species. Perhaps this is indicative of some level of specialisation of these two lineages.

The *Haemoproteus* tree identified seven clusters with the largest clusters comprising seven parasite lineages each (Figure 6). Twelve lineages did not cluster, of which six of those new lineages were detected for the first time in this study. Six previously reported lineages did not fall within the identified clusters and stood on their own. The *Haemoproteus* lineages appeared to be more diverse and less related than the *Plasmodium* and *Leucocytozoon* lineages. Most of the newly identified *Haemoproteus* lineages fell within the identified clusters. The lineage BUTVER01 (*Haemoproteus* sp.) was 96% similar to the lineage CATAUR01 in the MalAvi and GenBank databases (GenBank accession number MF953291), was described as *Haemoproteus catharti*, and was found in a Water thick-knee (*Burhinus vermiculatus*).

A comparison of the parasite lineages with closely described species in the Genbank and MalAvi databases revealed varying levels of diversity. The largest *Plasmodium* cluster grouped parasite lineages were unknown (unclassified). The second largest cluster aligned with *Plasmodium relictum* (96% to 100% similarity). *Plasmodium parahexamerium* and *Plasmodium homopolare* had similarity percentages ranging between 95% and 97% with the lineages PELSEP01, PELSEP02, and PELSEP04. *Plasmodium lutzi* and *Plasmodium matutinum* aligned with CRIMOZ03 with 97% and 98% similarity, respectively.

The *Leucocytozoon* lineages recorded in this study mainly represented two known *Leucocytozoon* species: *Leucocytozoon gentili* (22 lineages) and *Leucocytozoon californicus* (MalAvi ID FASPA02; three lineages). The *Haemoproteus* clusters were common and the most diverse represented the following species *Haemoproteus homobelopolskyi*, *Haemoproteus homominutus*, *Haemoproteus homopalloris*, *Haemoproteus homopicae*, *Haemoproteus lanii*, *Haemoproteus pallidus*, *Haemoproteus paranucleophilus*, *Haemoproteus pastoris*, *Haemoproteus sacharovi*, *Haemoproteus sanguinis*, and *Parahaemoproteus passeris*.

### 3.3. Host Specificity

The eight *Haemoproteus*, six *Plasmodium,* and four *Leucocytozoon* lineages infected at least two host species. The calculated S_TD__*_ values for all three parasite genera ranged between one and four and indicated the marked variation in host specificity. The parasite lineage that infected the highest number (*n* = 6) of bird species was RS4 (*Leucocytozoon* sp.; Table 3). For *Plasmodium*, three out of six lineages (RTSR1, SYBOR10, and TOCERY01) had an S_TD__*_ value larger than three, suggesting that there were generalists. The lineage RBQ22 from *Plasmodium*, with an S_TD__*_ value of three was also identified as a generalist, which was recorded in the Lesser striped Swallow (*Cecropis abyssinica*) and the Village Weaver (*Ploceus cucullatus*) who belong to the superfamilies Sylvioidea and Passeroidea, respectively. For *Haemoproteus*, most of the lineages were recorded as specialists. It was, however, noted that the lineages SPISEN02 and AFR173 (*Haemoproteus* sp.) had the highest S_TD__*_ values of four and three, respectively, and infected two unrelated species, indicative of a generalist lineage. For *Leucocytozoon*, two of the lineages (PASDIF03 and LAMCHA04) were generalists and the other two (REB7 and RS4) were specialists. In general, when considering only the lineages that infected two or more hosts in this study, *Plasmodium* and *Leucocytozoon* recorded the average S_TD__*_ values of 2.44 and 2.48, respectively, and were identified as generalists, whilst *Haemoproteus* had an average index of 1.76 indicating that the lineages found were specialists.

## 4. Discussion

Although studies on parasite–host interactions have greatly improved our understanding of the co-evolutionary relationship between a parasite and its host, a lot remains to be deciphered. Variations in the host specificity of parasite species (i.e., the diversity of the host species a parasite infects, ranging from specialist to generalist parasites) have shown to markedly differ from one region to another, driven by both the host’s traits and environmental factors (i.e., biomes and geographical barriers; [41]). In this study, we examined parasite prevalence, diversity, and host specificity in a community of avian haemosporidian parasites and explained these in relation to avian host diversity and sampling localities. Our findings, even though some samples were collected seven years ago, revealed a total avian haemosporidian infection prevalence of 28.41% which varied between parasite genera. There was also a relatively high degree of diversity in bird host species and parasite lineages, as well as a varying degree of host specificity among parasite lineages. Previous studies in other Afrotropical regions reported a higher avian haemosporidian infection prevalence than observed in this study, and a high level of parasite lineage diversity [20,42]. The authors in Illera et al. [43] also suggested that host richness might explain the variations in parasite prevalence and richness. In their study, *Plasmodium* prevalence and richness showed a negative correlation with host richness, whereas *Haemoproteus* and *Leucocytozoon* were positively correlated with host richness. The observed prevalence in this study could be explained by the dilution effect hypothesis [44,45,46,47]. This hypothesis posits that diverse ecosystems will limit disease transmission and spread, thus leading to a low disease prevalence [44]. In essence, it is in the interest of global public health to conserve biodiversity since rich and diverse communities will dilute the effect of parasites by lowering the risk of disease and reducing the rates of pathogen transmission. Hence, Civitello et al. [48] stressed that human-induced biodiversity loss would increase wildlife disease prevalence. Conversely, it is worth noting that a positive relationship (an amplification effect) between pathogen prevalence and biodiversity may occur [49]. The authors in Roiz et al. [50] for example, recorded a higher prevalence of the Usutu virus in areas with richer avian communities. This study did not exhaust all other elements, specifically assessing factors driving dilution, but perhaps it should be a topic to explore in another study.

Despite the relatively low prevalence recorded in this study compared to other Afrotropical regions, a high parasite lineage diversity was recorded here, corroborating previous findings and predictions from the region and globally [23,51,52,53]. The authors in Chaisi et al. [51] recorded an overall parasite prevalence of 68.82% in the Afrotropical terrestrial birds from 93 samples collected in South Africa (*n* = 76) and West Africa (N = 17), whilst Lutz et al. [20] recorded 79.1% from 532 birds sampled in Malawi. They also recorded an exceptionally high parasite diversity with 248 parasite cytochrome *b* lineages identified from 152 host species. The authors in Outlaw et al. [52], on the other hand, demonstrated that avian haemosporidian parasites exhibit a similar pattern of diversity to their hosts and suggested that parasites should be the most diverse in regions with the greatest proportions of endemic host species. Our study area was conducted in an Important Bird and Biodiversity Area (IBA), and, with the exception of four migrant bird species (Violet-backed Starling *Cinnyricinclus leucogaster*, Barn Swallow *Hirundo rustica*, Red-backed Shrike *Lanius collurio*, and the African Paradise Flycatcher *Terpsiphone viridis*), all other birds sampled during this study are endemic to the region, suggesting a link between host endemicity and the high diversity of the parasite lineages recorded (100 unique parasite lineages from 294 infected individuals). This assertion is further confirmed by the observed positive relationship between the bird and parasite diversity indices and confirmed by Wardjomto et al. [54]. In line with these findings, where parasite diversity is expected to increase with improved sampling, perhaps more bird samples and species diversity would unravel an elevated infection prevalence and diversity. Our current sample of the diversity (55 out of 490 species) represents approximately 11% of all bird species in this region. Nevertheless, the contribution of this study is significant since new avian haemosporidian lineages were found in 12 resident bird species, from the 55 bird species that were sampled.

The parasite diversity observed in this study can also be attributed to the diversity of the habitats, which sustain large populations of host species intertwined in a multitude of ecological and co-evolutionary processes with the vectors—we will further explore this in other studies. In essence, the ubiquitous nature of the *Plasmodium* vector (generally *Culex* mosquito, [24]) and the endemicity of human malaria in the region suggest that the habitat and environmental conditions are suitable for vector proliferation and disease prevalence. The vectors of the *Haemoproteus* and *Leucocytozoon* parasites, which require a semi-moist to arid habitat [24], may also find a suitable habitat in this Afrotropical region.

Most bird species sampled were infected by a single parasite lineage, indicating a high degree of host specificity in the area. Host specificity, in this case, could explain the low prevalence. The host-specific parasite lineage may infect a single or closely related species and thus affect their prevalence in the area, especially if the host distribution and numbers are limited. The longitudinal and latitudinal variation in the host species in the area is expected to limit the host distribution and therefore, affect parasite prevalence [55]. As observed here, sites inside Kruger National Park had a marginally higher infection (prevalence = 30%) than those outside (prevalence = 23%) the park, supporting the assertion that avian haemosporidian parasites are more prevalent in undisturbed areas than disturbed areas [11]. Although, urban and arid environments may hinder the development of competent vectors [11], thus keeping their prevalence relatively low in the region.

The host specificity index used in this study measured the average taxonomic distinctiveness weighted by the prevalence of the parasite in different hosts [7] and considered the haemoparasite species that infected two or more hosts for comparison purposes. This index is such that the lineages infected by one parasite only are assigned a value of zero or one or omitted altogether [7] with their host specificity classified as undetermined. The value of S_TD__*_ increases as the taxonomic distinctiveness between the high-prevalence hosts increases, although the effect on S_TD__*_ is greater with a more drastic change in the taxonomic distance than with changes in prevalence. The average observed host specificity index (S_TD__*_) values of 2.44 and 2.48 for *Plasmodium* and *Leucocytozoon,* respectively, suggest that these parasite genera were infecting birds that are distantly related (from different families or different orders) whilst *Haemoproteus* was infecting more closely related bird hosts (generally constrained to the family level). This finding is in line with those of [56] in Madagascar where the *Haemoproteus* lineages were mainly host-specific and the *Plasmodium* and *Leucocytozoon* lineages were generalist. The proximity of Madagascar to the mainland of Southern Africa could explain this similarity. Although the *Leucocytozoon* lineages are generally host specific [42], transitions from generalist to specialist and vice versa are common [3,57]. These transitions are suspected to be an evolutionary strategy driven by competition, climate change, and large-scale ecological perturbation [57,58]. By probing only the *Haemoproteus* Lineage SPISEN02 identified as a generalist (with the highest host specificity index of S_TD__*_ = 4), it was observed that it was present in two distantly related bird species (Red-headed Weaver: *Anaplectes rubriceps* and Laughing Dove) with equal prevalence value of 10% thus driving the value of S_TD__*_ up. Until now, this lineage had only been detected in Laughing Doves (evidenced by the MalAvi database; [37]). It is therefore possible that this lineage, although identified as a generalist, could be a host specific lineage. However, more data are needed to confirm this assertion. The *Leucocytozoon* lineages AFR173, RB7, and RS4 were found in birds of different orders and classes (i.e., highest taxonomic distance), confirming that these were generalist infections.

For the existing parasite lineages with undetermined host specificity in this study, the MalAvi database shed some light on their host specificity status. The lineage BUL2 (*Haemoproteus sanguinis*) was recorded infecting closely related bird species in the MalAvi database (Willow Warbler *Phylloscopus trochilus*, Dark-capped Bulbul *Pycnonotus tricolor*, White-spectacled Bulbul *Pycnonotus xanthopygos*, Malagasy Bulbul *Hypsipetes madagascariensis*, and African red-eyed Bulbul *Pycnonotus nigricans*) mainly from the African continent and predominantly infecting the Dark-capped Bulbul (*Pycnonotus tricholor*) observed in this study. The lineage BUL2 could therefore be classified as host-specific, infecting closely related species [37]. The lineages CRECRI01, CRECIN01, and CRECIN02 are also host-specific infecting only the Wattled Starling (*Creatophora cinerea*). In essence, except for SPISEN01 (infecting *Passer domesticus* and *Streptopelia senegalensis*), nearly all the *Haemoproteus* lineages recorded in this study were host-specific. Similarly, except for the parasite lineages recorded for the first time in this species, the existing *Plasmodium* lineages (AEMO01, COLL7, LINOLI01, MELMEL06, MALNI02, RBQ22, RTSR1, and SYBOR10) appeared to be generalists. Furthermore, except for the lineages recorded for the first time in this study, all existing MalAvi *Leucocytozoon* lineages recorded in this study (AFR161, AFR164, AFR173, EUPHOR02, PASDIF03, REB7, RECOB3, RS4, SYBOR06, and WCH2) also appeared to be generalists.

The relationship between the parasite lineages and infection patterns confirmed the findings of the host specificity pattern of the lineages recovered in this study, although exceptions were observed. The parasite lineages from the largest cluster of the *Haemoproteus* genus were found to be infecting other bird species in the MalAvi database as well, suggesting that parasite lineages from this cluster are generalists. A high level of parasite lineage endemicity was observed among the parasite lineages recorded in this study. This was evident in the high number of new parasite lineages recorded and the lack of described, closely related, parasite species for a large number of the lineages recorded in this study (26 lineages in total). Six parasite lineages (COLL7, LINOLI01, PLOCUC08, PLOCUC13, RBQ22, and TOCLEU01) were closely related to *Plasmodium relictum*, the causal agent of avian malaria responsible for the mortality and extinction of several Hawaiian bird species [59]. It is, however, noted that the level of pathology may differ for closely related parasite lineages and strains in different bird species [37]. Further studies may be necessary to provide clarity on the extent of the effect of closely related parasite strains on avian hosts.

## 5. Conclusions

This study is the first large-scale study to molecularly describe avian haemosporidian parasites in the Afrotropical conservation area around Kruger National Park in South Africa. A low parasite prevalence was observed in this study, but a high parasite diversity and a large number of new parasite lineages were identified which will contribute to enriching the existing avian malaria and associated haemosporidian parasites database (MalAvi). The *Haemoproteus* lineages were generally specialist whilst the *Plasmodium* and *Leucocytozoon* lineages were generalist, resulting in a marked phylogenetic structure. The observed positive relationship between avian host diversity and parasite diversity is indicative of an amplification effect. These findings provide the opportunity to test new hypotheses to improve our understanding of host–parasite co-evolution, the drivers of parasite infection, the prevalence and diversity of parasites in a fairly natural setting, as well as the factors that lead to host specialisation and generalisation.

## Figures and Tables

**Table 1:** Summary of infections per parasite genus in the lowveld region of South Africa.

	*Haemoproteus*	*Plasmodium*	*Leucocytozoon*	Total
No of infections detected	180	48	96	294
Prevalence (%)	17.39	4.64	9.28	28.41
Number of lineages	45	26	29	100
New identified Lineages	23	16	17	56
Existing MalAvi Lineages	22	10	12	44

**Table 2:** A comparison of bird and parasite lineage species richness (*S_H_* and *S_P_*) and diversity indices (*H_H_* an *H_P_*) calculated for each sampling site (inside vs. outside Kruger National Park).

			Host Species		Parasite Lineage	
Location	Sampling Sites	Coordinates	*S_H_*	*H_H_*	*S_P_*	*H_P_*
Outside Kruger National Park	Acornhoek	31.041156° −24.587340°	10	1.84	13	2.46
	Hazyview	31.185619° −25.032365°	4	1.13	1	0
	Malelane	31.574146° −25.467964°	11	1.73	7	1.91
	Mkhuhlu	31.241542° −24.995130°	15	2.13	22	2.72
Inside Kruger National Park	Phalaborwa	31.169120° −23.937940°	16	2.51	14	2.56
	Satara	31.774039° −24.397732°	19	2.08	21	2.77
	Shangoni	30.975002° −23.239999°	7	1.89	4	1.39
	Shingwedzi	31.425900° −23.113545°	11	1.82	14	2.34
	Skukuza	31.603911° −24.996356°	36	2.46	46	3.28

**Table 3:** Number of hosts infected by each parasite lineage and their corresponding specificity index (S_TD__*_). Only lineages that infected more than one host are presented.

Genus	Lineage	Host Infected	S_TD__*_	Host Specificity
*Haemoproteus*	AFR041	2	1	Specialist
	AFR076	2	1	Specialist
	AFR084	2	2	Specialist
	PAMEL01	3	1.98	Specialist
	SPISEN02	2	4	Generalist
	VILWE2	4	1.32	Specialist
	VIMWE1	3	1	Specialist
	AFR173	2	3	Generalist
*Leucocytozoon*	PASDIF03	2	1	Specialist
	REB7	4	3	Generalist
	RS4	6	2.90	Generalist
	LAMCHA04	2	1	Specialist
*Plasmodium*	LAMCHA05	2	1	Specialist
	MALNI02	2	1	Specialist
	RBQ22	2	3	Generalist
	RTSR1	3	3.98	Generalist
	SYBOR10	4	3.73	Generalist
	TOCERY01	3	3.37	Generalist

Haplotypes were specific to avian families.

## Data Availability

Data for this study are freely available from the South African Foundational Biodiversity Information Programme (FBIP) repository and are deposited at the following link: https://figshare.com/articles/dataset/Data_01_12_2020_xlsx/13317293 (assessed on the 1 December 2020). Accession numbers of the new parasite lineages detected are available in the GenBank and MalAvi databases.

## Supplementary Materials

The following supporting information can be downloaded at: https://www.mdpi.com/article/10.3390/ani14192906/s1, Table S1: summary of all birds sampled, as well as the prevalence and corresponding parasite lineages recorded in each infected bird host. Title headings: *n* = sample size; H = *Haemoproteus* spp.; P = *Plasmodium* spp.; and L = *Leucocytozoon*. The bold lineages indicate new lineages. The numbers in the grey column indicate the sample sizes of the bird(s) infected by a lineage for that species.
