# Supplementary material for: Diversity and Host Specificity of Avian Haemosporidians in an Afrotropical Conservation Region
Source: Animals (Basel). 2024 Oct 9;14(19):2906. doi: 10.3390/ani14192906 (PMC11475415; doi:10.3390/ani14192906)
Supplement: Supplementary file 1 [file animals-14-02906-s001.zip › animals-3145774-supplementary.pdf]

Supplementary Table

Table S1. Summary of all bird sampled, prevalence and the corresponding parasite lineages recorded in each infected bird host. Title headings *n* = sample size; *H* = *Haemoproteus* spp; *P* = *Plasmodium* spp; *L* = *Leucocytozoon*. Bold lineages indicate new lineages. Numbers in grey column indicate sample sizes of bird(s) infected by lineage for that species.

Species	*n*	Infected	Prevalence (%)	*H*	*P*	*L*	Lineages	
Acridotheres tristis	2	0	0	0	0	0		
Actophilornis africanus	5	0	0	0	0	0		
Alopochen aegyptiaca	3	0	0	0	0	0		
Amaurornis flavirostris	1	0	0	0	0	0		
Anaplectes rubriceps	10	6	60	4	0	2	AFR173	1
							ANARUB02	1
							ANARUB03	3
							SPISEN02	1
Bostrychia hagedash	1	1	100	1	0	0	**BOSHAG01**	1
Bradypterus baboecala	1	1	100	1	0	0	**BRABAB01**	1
Buphagus erythrorhynchus	30	14	43	0	0	13	**BUPERY01**	4
							REB7	3
							RECOB3	5
							RS4	2
Burhinus vermiculatus	2	2	100	2	0	0	BUTVER01	2
Cecropis abyssinica	4	2	50	1	1	0	RBQ22	2
Cinnyricinclus leucogaster	4	0	0	0	0	0		
Colius striatus	6	0	0	0	0	0		
Cossypha heuglini	1	0	0	0	0	0		
Creatophora cinerea	38	16	42	13	3	0	AFR084	1
							**CRECIN01**	1
							CRECIN02	1
							CRECRI01	10
							RTSR1	1
							**TOCERY01**	2
Crithagra mozambica	25	17	56	9	1	7	AFR072	1
							AFR073	4
							AFR161	1
							**CRIMOZ02**	2
							**CRIMOZ03**	1
							**CRIMOZ04**	1
							REB7	5
							SPISAL01	1
							SYBOR06	1
Dicrurus adsimilis	14	2	29	2	0	0	**DICADS01**	2
Emberiza flaviventris	1	1	100	1	0	0	**EMBFLA01**	1
Euplectes orix	9	0	0	0	0	0		
Gymnoris superciliaris	1	1	100	1	0	0	PAMEL01	1
Halcyon albiventris	5	1	20	1	0	0	**HALALB01**	1
Hirundo rustica	28	1	7	1	0	0	HIRUS05	1
Hirundo smithii	22	4	18	4	0	0	Unidentified	

Species	*n*	Infected	Prevalence (%)	*H*	*P*	*L*	Lineages	
Lamprotornis australis	1	1	100	0	1	0	LAMCHA05	1
lamprotornis chalybaeus	191	64	34	45	18	2	AFR041<br>AFR076<br>AFR084<br>LAMCHA01<br>LAMCHA02<br>LAMCHA03<br>LAMCHA04<br>LAMCHA05<br>LUME1<br>RS4<br>RTSR1<br>SYBOR10	
Lamprotornis nitens	15	5	33	5	0	0	AFR041<br>AFR076<br>LAMCHA04	
Lanius collurio	1	1	100	1	0	0	RBS5	
Leptoptilos crumeniferus	1	1	100	0	1	0	RTSR1	
Lybius torquatus	3	0	0	0	0	0		
Melaniparus niger	1	1	100	1	0	1	RS4	
Merops bullockoides	6	0	0	0	0	0		
Motacilla aguimp	7	0	0	0	0	0		
Passer diffusus	31	20	65	14	1	10	AFR042<br>AFR173<br>PAMEL01<br>PAMEL02<br>PASDIF01<br>PASDIF02<br>PASDIF03<br>PASDIF04<br>PASDIF05<br>PASDIF06<br>PASDIF07<br>RS4	
Passer domesticus	84	18	21	3	0	16	AFR164<br>PADOM39<br>PAMEL01<br>PASDIF03<br>REB7<br>RS4<br>VILWE2	
Peliperdix sephaena	10	5	50	0	3	3	PELSEP01<br>PELSEP02<br>PELSEP03<br>PELSEP04	
Phoeniculus purpureus	1	0	0	0	0	0		
Ploceus capensis	7	4	57	3	1	0	LINOLI01<br>MALNI02<br>VILWE04<br>VILWE2	
Ploceus cucullatus	69	38	55	18	15	8	AEMO01<br>COLL7<br>LINOLI01<br>MALNI02<br>PLOCUC06	

Species	*n*	Infected	Prevalence (%)	*H*	*P*	*L*	Lineages
							PLOCUC07<br>PLOCUC08<br>PLOCUC09<br>PLOCUC11<br>PLOCUC12<br>PLOCUC13<br>RBQ22<br>REB7<br>RS4<br>SYBOR10<br>VILWE2<br>VIMWE1<br>WCH2<br>WW3
Ploceus intermedius	23	4	17	4	0	0	VILWE2<br>VIMWE1
Ploceus velatus	8	3	38	2	1	0	MELMEL06<br>PLOVEL02<br>VIMWE1
Prinia subflava	7	1	14	1	0	0	PRISUB01
Prionops plumatus	5	0	0	0	0	0	
Pycnonotus tricolor	17	8	47	4	1	7	AFR223<br>BUL2<br>PYCBAR03<br>PYCTRI01<br>PYCTRI02<br>PYCTRI03<br>PYCTRI04
Pytilia melba	2	0	0	0	0	0	
Quelea quelea	65	1	2	1	0	0	Unidentified
Spermestes cucullata	5	0	0	0	0	0	
Spilopelia senegalensis	102	35	34	22	0	20	SPISEN01<br>SPISEN02<br>SPISEN04<br>SPISEN05<br>SPISEN06
Streptopelia decipiens	54	2	4	2	0	0	STRTUR03
Streptopelia semitorquata	8	3	38	3	0	1	STRSEM01
Terpsiphone viridis	9	1	11	1	0	1	EUPHOR02<br>TERUF01
Tockus erythrorhynchus	40	3	5	1	1	0	SYBOR10
Tockus leucomelas	30	1	3	1	0	0	TOCLEU01
Trachyphonus vaillantii	5	2	40	2	0	1	AFR002<br>CIAE02
Turdoides jardineii	6	3	50	0	0	3	TURJAR02<br>TURJAR03<br>TURJAR04<br>TURJAR05
Tychaedon quadrivirgata	1	1	100	1	0	1	TYCQUA01<br>TYCQUA02
Uraeginthus angolensis	7	1	14	1	0	0	URANG01
**Total**	**1035**	**294**		**180**	**48**	**96**	
